# Degradation of fipronil by *Stenotrophomonasacidaminiphila* isolated from rhizospheric soil of *Zea mays*

**DOI:** 10.1007/s13205-015-0354-x

**Published:** 2016-02-08

**Authors:** Shivani Uniyal, Rashmi Paliwal, R. K. Sharma, J. P. N. Rai

**Affiliations:** 1 Department of Environmental science, Swami Ramtirth campus Badshahithaul, Hemwati Nandan Bahuguna Garhwal University, Srinagar Garhwal, Uttarakhand India; 2 Ecotechnology laboratory, Department of Environmental Science, G. B. Pant University of Agriculture and Technology, Pantnagar, India; 3 G. B. Pant Institute of Himalayan Environment and Development, Himachal Unit, Mohal-Kullu, Himachal Pradesh India

**Keywords:** Fipronil, *Stenotrophomonas acidaminiphila*, Box–Behnken design, Bioremediation, Soil

## Abstract

Fipronil is a widely used insecticide in agriculture and can cause potential health hazards to non-target soil invertebrates and nearby aquatic systems. In the present study, a fipronil degrading bacterium was isolated from fipronil contaminated soil, i.e. rhizospheric zone of *Zea mays*. Morphological, biochemical and molecular characterization of strain indicated that it clearly belongs to *Stenotrophomonas acidaminiphila* (accession no. KJ396942). A three-factor Box–Behnken experimental design combined with response surface modeling was employed to predict the optimum conditions for fipronil degradation. The optimum pH, temperature and total inocula biomass for the degradation of fipronil were 7.5, 35 °C and 0.175 g L^−1^, respectively. The bacterial strain was able to metabolize 25 mg L^−1^ fipronil with 86.14 % degradation in Dorn’s broth medium under optimum conditions. Metabolites formed as a result of fipronil degradation were characterized with gas liquid chromatograph. A novel fipronil degradation pathway was proposed for *S.* *acidaminiphila* on the basis of metabolites formed. Non-sterilized soil inoculated with *S.* *acidaminiphila* was found to follow first order kinetics with a rate constant of 0.046 d^−1^. Fipronil sulfone, sulfide and amide were formed as the metabolites and were degraded below the quantifiable limit after 90 days of time period. Given the high fipronil degradation observed in the present study, *S.* *acidaminiphila* may have potential for use in bioremediation of fipronil contaminated soils.

## Introduction

The widespread use of increasing number of pesticides in agriculture has acquired great importance due to direct and indirect hazards to human health and environment. Globally, about 3 × 10^9^ kg of pesticides is applied annually (Pan-UK 2003); however, a large amount of applied pesticides often never reach their intended target due to their degradation, volatilization and leaching, leading to serious ecological problems (Chen et al. 2009; Chevillard et al. 2012). Fipronil [5-Amino-3-cyano-1-(2, 6-dichloro 4 trifluoromethylphenyl)-4-trifluoromethyl sulfinyl pyrazole] is a phenyl pyrazole insecticide first synthesized by Rhone Poulenc Ag Company (now Bayer Crop Science) in 1987, introduced for use in 1993 and registered in the U.S. in 1996 (Tomlin 2000; Ware 2000; Tingle et al. 2003). It is one of the most persistent, lipophilic and toxic insecticides licensed for use since dieldrin, lindane and DDT (Mohapatra et al. 2010). It controls a broad spectrum of insects such as rice stem borer, leaf folder, cockroaches, mosquitoes, locust, ticks and fleas at both their larval and adult stages (Chanton et al. 2001; Aajoud et al. 2003). Fipronil is a “new generation” insecticide as its mode of action does not follow the common biochemical pathways of classical insecticides such as, pyrethroids (sodium channel blockers), organophosphates, and carbamate (cholinesterase inhibitors) to which some insects have developed resistance (Aajoud et al. 2003). Fipronil elicits its toxicity by blocking the GABA-gated chloride channel in the nervous system, resulting in a disruption of neuron signalling and eventually shutdown of the central nervous system (Ecobichon 1996). Fipronil degradation results in the formation of metabolites viz. sulfide, sulfone, amide and desulfinyl (Gunasekara et al. 2007). The half-life of fipronil in soil varies greatly, ranging from 3 days to 7 months.

Bioremediation processes are considered to be cost effective tool for the detoxification of xenobiotics as microbes play an important role in removing toxic substances from the environment (Li et al. 2012; Paliwal et al. 2015a). Microbes have been used in remediation of pesticide contaminated environment (Ramanathan and Lalithakumari 1999; Phugare and Jadhav 2014; Sidhu et al. 2014). Previously, microbial degradation of fipronil was reported in literature (Zhu et al. 2004; Masutti and Mermut 2007; Lin et al. 2008) but very few reports are available on isolation and characterization of specific bacteria, able to degrade fipronil. For instance, *Bacillus firmus* and *Bacillus thuringiensis* were found to be competent in the rapid degradation of fipronil (Mandal et al. 2013, 2014). In addition *Paracoccus* sp. was also found to degrade fipronil (Kumar et al. 2012). Rate of fipronil degradation was reported to affect by many environmental factors including temperature, pH, moisture, formulation, soil composition and biotic factors (Zhu et al. 2004; Masutti and Mermut 2007). For effective biodegradation therefore, it is essential to optimize the process as it makes the technology strong by improving the microbial activities (Prakasham et al. 2005; Paliwal et al. 2015b). However, in none of the previous study, optimization of environmental factors, affecting the rate of fipronil degradation by microbes was conducted.

The aim of this study was therefore to isolate and characterize bacteria, capable of degrading fipronil. Present study reports fipronil degradation by *Stenotrophomonas acidaminiphila* for the first time. Besides this, response surface methodology (RSM) based on the Box–Behnken design was used to determine the optimum environmental conditions for growth and fipronil degradation by *S. acidaminiphila*. Metabolites formed as a result of degradation were identified and a novel metabolic pathway for fipronil degradation by *S. acidaminiphila* was proposed. The degradation rate of fipronil in soil inoculated with *S. acidaminiphila*, based on the kinetics of degradation was studied. This paper also highlights potential use of pure cultured *S. acidaminiphila* cells for the remediation of fipronil contaminated soils.

## Materials and methods

### Chemicals and media

Technical grade fipronil (Regent 0.3 % G, purity, 97.5 %) obtained from Bayer Crop Science Ltd, India was used. Dorn’s broth media used for the isolation of bacteria contained the following (g L^−1^): Na_2_HPO_4_·12H_2_O 3.0 g, KH_2_PO_4_ 1.0 g, (NH_4_)SO_4_ 1.0 g, MgSO_4_·7H_2_O 10.0 g, CaCl_2_·2H_2_O 2.0 g, MnSo_4_·H_2_O 3.0 g, FeSO_4_·7H_2_O 0.2 g, Ammonium ferric citrate 0.01 g, Yeast extract 0.1 g, Distilled water, pH 7.0 and was sterilized at 121 °C for 20 min (Kumar et al. 2012). Dorn’s broth media was amended with fipronil (25 mg L^−1^) as a sole source of carbon and nitrogen. Solid media plates were prepared by adding 2 % agar into above liquid media.

### Isolation and identification of fipronil degrading strain

Soil samples with previous history of fipronil application were collected from the rhizospheric zone (0–20 cm) of *Zea mays* plantation, situated at Crop Research Centre of the G.B.P.U.A.T. Pantnagar. Fipronil degrading strains were isolated from soil using the enrichment method of Wang et al. (2013). Strain encoded as S1 was selected on the basis of its highest ability for fipronil degradation and investigated for morphological characteristics and biochemical properties by API 20 NE system (Analytical Profile Index, France). Strain was identified by partial sequencing of 16S rRNA. Amplification of the 16S rRNA gene of the bacterial isolate was performed with universal bacterial primers 8f–1512r (Karpouzas et al. 2010). PCR was performed under the following conditions: 30 cycles of denaturation at 94 °C (1 min), annealing at 55 °C (1 min) and extension at 72 °C (1 min). PCR products were purified and sequenced by Chromus Biotech Ltd. Banglore, India. Identification was carried out on the basis of 16S rRNA homology between the query and reference sequences available at GenBank using BLAST algorithm of the National Centre for Biotechnology Information (NCBI) database at www.ncbi.nlm.nih.gov/BLAST. Phylogenetic analysis was done using the neighbor-joining method with bootstrap values calculated from 1000 replicate runs, with MEGA 5.1 software.

### Optimization of the fipronil degrading conditions

A Box-Behnken design was performed in order to study the effects of the incubation temperature (25–45 °C), pH (5–10) and total inocula biomass (0.10–0.25 g L^−1^) on fipronil biodegradation by single-factor experiments. Response surfaces were drawn as a function of two independent variables, while the third was set at a fixed value. Meanwhile, fipronil degradation was investigated to determine the effects of the three factors, namely, incubation temperature, pH and total inocula biomass. Response surface methodology (RSM) based on the Box–Behnken design was used to investigate the influence of interactive effects of the selected parameters on fipronil degradation by strain S1. Box–Behnken design with twelve factorial points and three replicates at the center point was employed in this experiment. A total of 15 runs were used for optimizing the range and levels of chosen variables. Second-order polynomial equation was assumed for predicted response (Eq. 1):

1 $$\beta_{0} + \mathop \sum \limits_{i = 1}^{k} \beta_{i} X_{i} + \mathop \sum \limits_{i = 1}^{j - 1} \mathop \sum \limits_{j = 1}^{k} \beta_{ij} X_{i} X_{j} + \mathop \sum \limits_{i = 1}^{k} \beta_{ii} X_{i}^{2}$$

X_*i*_ and X_*j*_ are variables, *β*_*o*_ is the constant, *β*_*i*_ is the linear coefficient, *β*_*ii*_ is the quadratic coefficient, and *β*_*ij*_ is the interaction coefficient. Design Expert 8.0.0 (trial version, Stat Ease Inc., Minneapolis, USA) computer program was used for determination of the coefficients of Eq. (1) by regression analysis of the experimental data.

### Degradation study

Logarithmic phase cultures of strain S1 in Dorn’s broth media were harvested by centrifugation and washed twice with deionised water. Cell pellets (0.175 g L^−1^) were mixed in sterilized Dorn’s broth media, and maintained at approximately 1.5 optical densities at 600 nm with 25 mg L^−1^ of fipronil as a sole source of carbon and energy. UV–VIS Spectrophotometer (Varian 50 Bio, USA) was employed for monitoring cell growth. Bacterial cultures were incubated at 35 °C, pH 7.5 and 120 rpm on a rotary shaker. Samples were collected from the cultures at an interval of 14 days. 5 mL of samples were centrifuged at 12,000*g* for 10 min, extracted with hexane (30 mL). Control flask, without culture was also maintained at the same condition. All of the degradation experiments were performed in triplicates. Samples were analyzed by gas liquid chromatograph (GLC) to evaluate residual concentration of fipronil and its metabolites.

### Fipronil degradation in soil inoculated with strain S1

Fipronil degradation by strain S1 was performed in sandy loam soil samples having pH 7.2, organic carbon 0.78 %, available N 196.75 kg ha^−1^, available P 18.24 kg ha^−1^ and available K 162.37 kg ha^−1^. Fipronil degradation study was performed in two different conditions viz. non-sterilized soil without inoculation (control) and non-sterilized soil inoculated with strain S1. Soil samples were fortified with fipronil at the rate of 50 mg kg^−1^ and then inoculated along with 45  ×  10^7^ microbe cells. Each treatment was replicated thrice. Soil samples were incubated at 30 °C in dark and soil moisture was maintained at 40 % (w/w of dry weight of soil). Soil samples were collected and extracted with acetonitrile-acetone at regular time interval as per method described by Mohpatra et al. (2010). A representative 50 g soil sample was extracted with 100 mL 7:3 (v/v) acetonitrile-acetone followed by its filtration under vacuum through a Buchner funnel. The combined extracts were collected after washing the container and the filter cakes twice with 100 mL of the solvent mixture. The acetonitrile-acetone fraction was concentrated and the residues obtained were diluted with 30 mL saturated sodium chloride solution. The contents were partitioned three times into 50 mL 1:1 (v/v) hexane–ethyl acetate. The hexane–ethyl acetate fraction was concentrated to 5 mL by drying over anhydrous sodium sulphate. The extracts were filtered by passing through column with florosil as the adsorbent and analyzed by GLC.

Analytical method was validated by fortification of fipronil and all its metabolites in soil at the rate of 0.01, 0.1 and 1.0 mg kg^−1^. Recovery of fipronil and its metabolites were in the range of 94–98 %. Fipronil and its metabolites was analyzed using GLC Hewlett-Packard 5890 series II equipped with electron capture detector (ECD) U.H.P. grade. A capillary column, equity 5 was used and flow rate of Helium as a carrier gas was maintained at 1 mL min^−1^. Following temperature programming was used: an oven was initially held at 100 °C for 0.5 min, and then for 3 min. Ion source and interface temperature were 230 and 260 °C, respectively. Standards of fipronil and all its metabolites were run under identical operating conditions for the identification and quantification of compounds formed after degradation.

### Data analysis

All of the experiments were performed in triplicates. The data were evaluated using analysis of variance (ANOVA) and the statistical significance of difference among the treatments was analyzed by SPSS statistical package (Statgraphics Plus V. 11). Means with significant differences were expressed at 0.05 probability levels.

## Results and discussion

### Isolation and identification of fipronil degrading strain

**Fig. 1:**
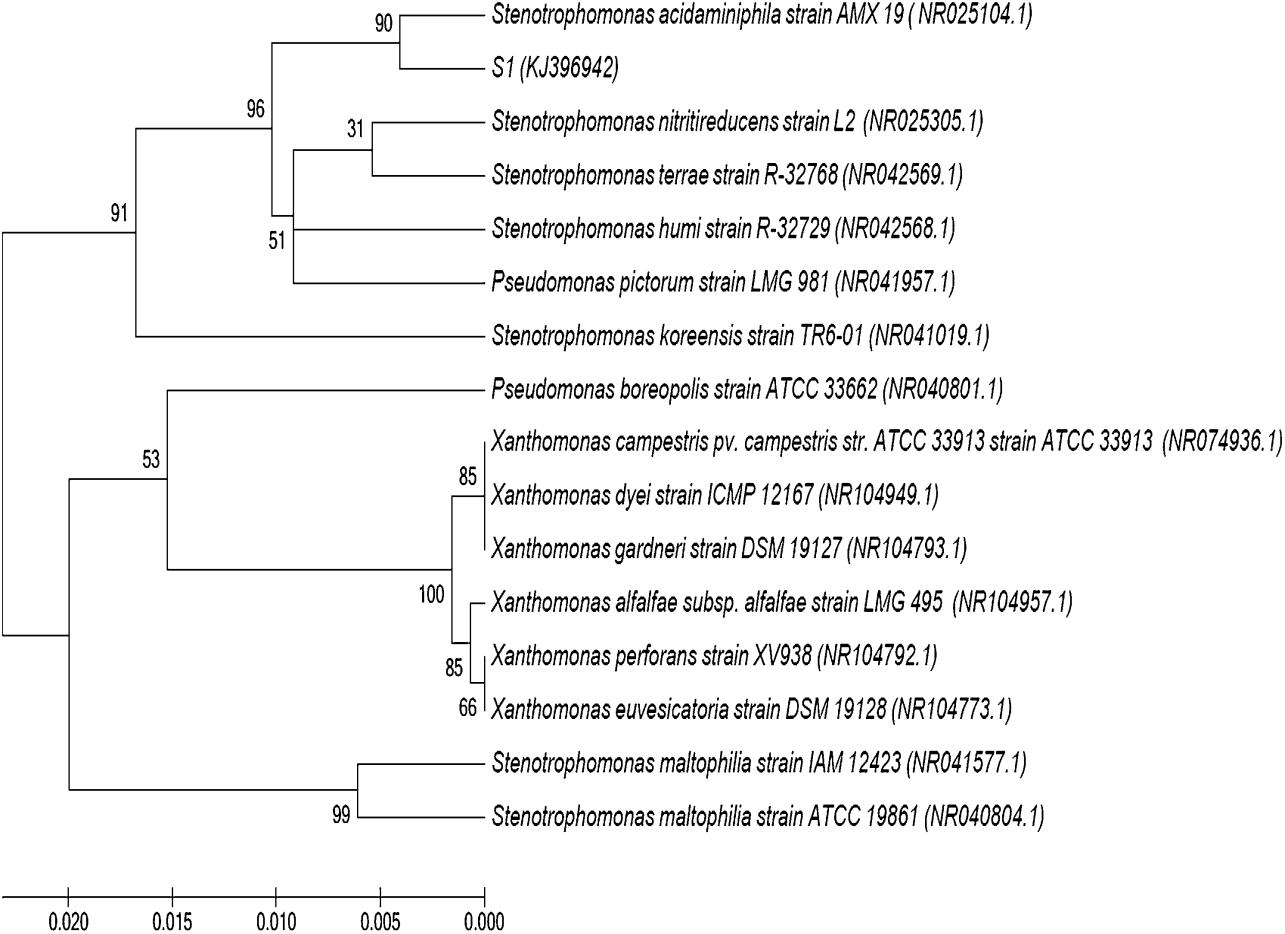
Phylogenetic tree of strain S1 constructed by the neighbor-joining method based on nucleotide sequences of the partial *16S rRNA* genes. The number at the nodes represents percentage bootstrap value of 1000 replicates

**Fig. 2:**
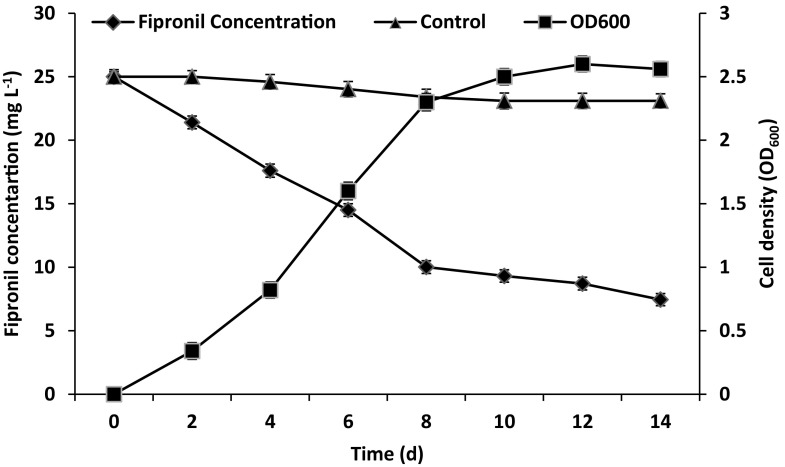
Growth of bacterial strain S1 in Dorn’s broth medium supplemented with fipronil (25 mg L^−1^) as a sole carbon source. Error bars represent the standard deviation within 5 % of the mean

An indigenous bacterial strain capable of utilizing fipronil as a sole carbon and energy source was eventually isolated from the fipronil contaminated soil. Strain S1 was a gram negative, rod shaped bacterium. Strain S1 showed positive enzymatic reactions for H_2_S production, oxidase, catalase with assimilation of lysine, ornithine, d-glucose, d-fructose, mannose and arabinose (Table 1). Sugar assimilation efficiency of *Stenotrophomonas* sp. has been reported by Uniyal et al. (2013). Results of 16SrRNA sequencing using universal primers with data bank sequences (GenBank) indicated 100 % similarity of strain S1 with *Stenotrophomonas acidaminiphila*. The resultant phylogenetic tree depicts the phylogenetic relationship of this isolate with already reported species in GenBank (Fig. 1). The 16S rRNA gene sequence of strain S1 was deposited in the GenBank nucleotide sequence databases under accession no. KJ396942. Growth profile of bacterial isolate S1 showed most effective growth of bacterial isolate within 8 days of incubation; correspondingly, fipronil concentration sharply declined within same time period and finally degraded up to 70.2 % of the initial concentration after 14 days of incubation period (Fig. 2). No change in OD 600 was observed for non-inoculated control within 14 days of incubation period. Results indicated that isolated strain can efficiently utilize fipronil as a sole carbon and energy source. Several bacterial species such as *Paracoccus* sp. (Kumar et al. 2012), *B. firmus* (Mandal et al. 2013) and *B. thuringiensis* (Mandal et al. 2014) are reported to utilize fipronil as a sole source of carbon and energy (Table 2).

**Table 1:** Biochemical characteristics for fipronil degrading soil isolate S1

Characteristics	**S1**
Morphological	
Colony color	Pale yellow
Gram nature	−
Cell morphology	Rod
Biochemical test	
H_2_S production	+
Urea	−
Oxidase	+
Catalase	+
Phenylalanine deamination	−
Nitrate reduction	−
Lysine	+
Ornithine	+
Utilization of C-Sources	
d-Glucose	+
d-Fructose	+
Mannose	+
Adonitol	−
Arabinose	+
Citrate	−
Lactose	−
Sorbitol	−

+ positive, − negative

**Table 2:** Fipronil degradation by bacteria in soil

Bacteria employed	Initial dose	Time taken to achieve fipronil degradation below detectable limit (days)	Reference
*Stenotrophomonas acidaminiphila*	50.0 mg kg^−1^	90	Present study
*Bacillus firmus*	1.5 mg kg^−1^	35	Mandal et al. (2014)
*Bacillus thuringiensis*	1.5 mg kg^−1^	35	Mandal et al. (2013)
*Paracoccus* sp.	80.0 µg kg^−1^	60	Kumar et al. (2012)

### Optimization of the fipronil-degrading conditions by strain S1

The optimum ranges of three significant factors (temperature 25–45 °C; pH 5–10; biomass amount 0.1–0.25 g L^−1^) were tested for fipronil degradation by single-factorial experiments. The experimental and predicted values of degradation by a complete three-factor-three level factorial experimental design, with three replications at the central points, are represented in Table 3. The quadratic model was employed for response of fipronil degradation. Subsequently the obtained results were fitted with the second-order polynomial equation (Eq. 2):

**Table 3:** Box–Behnken design matrix along with the experimental and predicted values of fipronil degradation

	Factor 1	Factor 2	Factor 3	Predicted response	Experimental response
Run	A:X1	B:X2	C:X3	R1	R2
	°C	pH	g/l	Percent degradation	Percent degradation
1	35	10.0	0.100	46.90	51.20
2	45	5.0	0.175	37.30	38.14
3	45	7.5	0.250	56.50	57.50
4	35	5.0	0.250	78.80	80.17
5	45	7.5	0.100	44.16	44.29
6	35	10.0	0.250	57.10	57.96
7	35	7.5	0.175	82.70	83.88
8	35	7.5	0.175	83.00	85.21
9	35	7.5	0.175	85.30	86.14
10	25	5.0	0.175	30.80	31.76
11	25	7.5	0.100	56.20	56.56
12	25	10.0	0.175	30.60	31.50
13	25	7.5	0.250	72.20	73.89
14	35	5.0	0.100	50.60	52.30
15	45	10.0	0.175	0	0

2 $$\begin{aligned} Y = - 634.642 + 23.375X_{1} + 83.528 X_{2} + 107.652X_{3} - 0.371X_{1} X_{2} - 1.22 X_{1} X_{3} - 24 X_{2} X_{3} - 0.300X_{1}^{2} - 4.634 X_{2}^{2} + 646.519X_{3}^{2} \hfill \\ \end{aligned}$$

*Y* is the predicted value of fipronil degraded by strain S1; *X*_*1*_, *X*_*2*_ and *X*_*3*_ are the coded values of the temperature, media pH and inocula biomass, respectively.

**Fig. 3:**
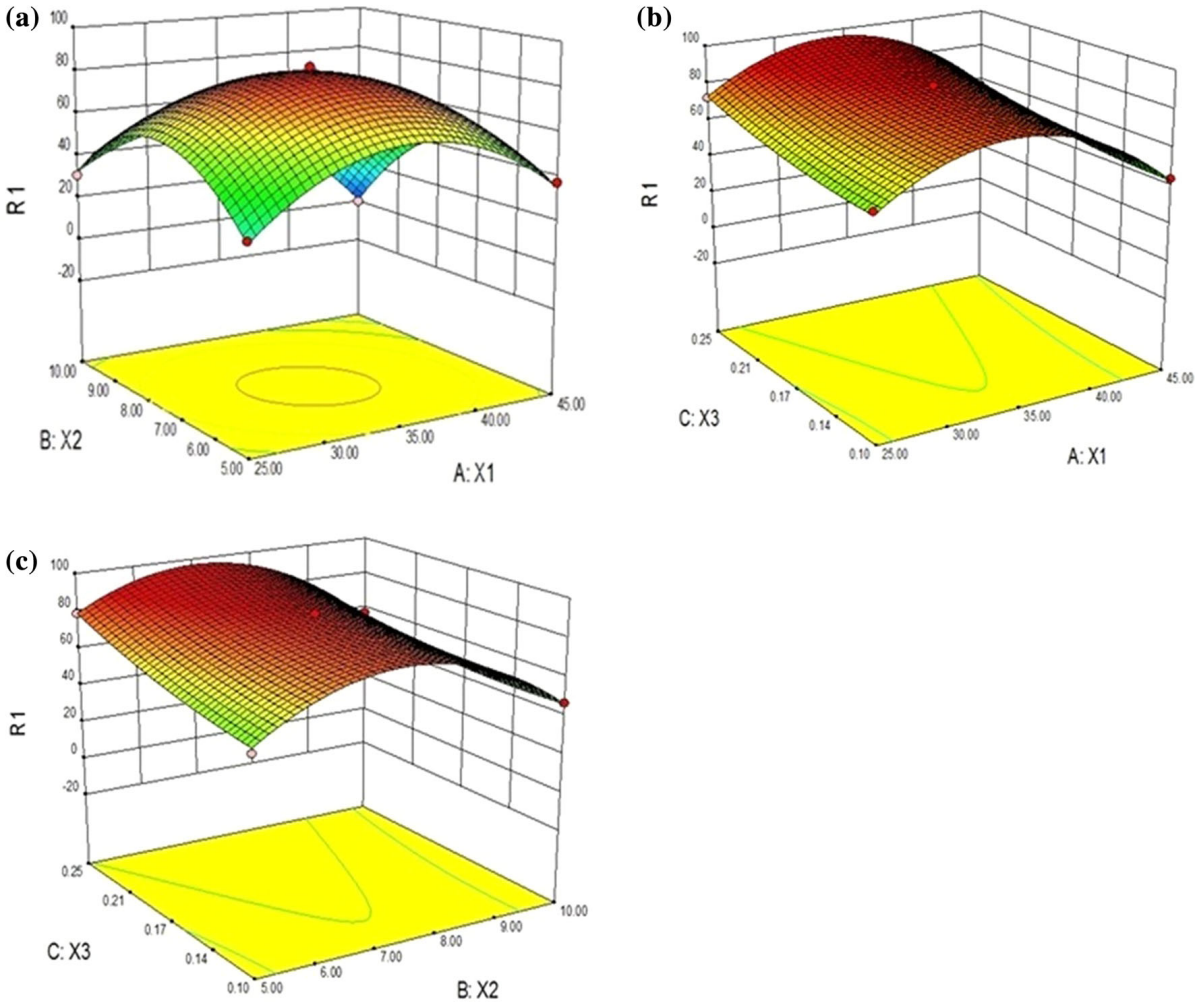
Response surface curves described by the model *Y*, which represents the effect of interactions between **a** temperature (*X*_*1*_) and pH (*X*_*2*_) **b** total biomass inocula (*X*_*3*_) and temperature (*X*_*1*_) **c** total biomass inocula (*X3*) and pH (*X2*) on fipronil degradation

The significant *F*-value (121.46) and the non-significant lack-of fit (0.16) value through the analysis of variance of the data imply that the quadratic model is highly significant (Table 4). Thus, the quadratic polynomial model, used in the present experiment adequately represented the actual relationship between response and variables. The *P* value serves as a tool for checking the significance of each of the coefficients, which also indicates the interaction strength of each parameter (Acikel et al. 2010). *P* values pointed that the linear and square terms of temperature (*X*_*1*_) and pH (*X*_*2*_) values were highly significant (*p* < 0.01). However, the interaction effects of linear and square terms of inocula biomass amount (*X*_*3*_) showed insignificant effects (*p* > 0.05) on the fipronil degradation by strain S1. The coefficient of determination (*R* ^*2*^) of the model was 0.995, which further indicated a good agreement between the experimental and predicted values for fipronil degradation. The value of the coefficient of variation (CV = 5.00 %), which also revealed adequacy of the model. Using RSM, the three-dimensional response surface plots of fipronil degradation were presented as a function of independent variables (Fig. 3). An elliptical response surface was observed with maximum response near centre point indicating that the interaction between corresponding variables is significant. At higher temperature and pH, fipronil degradation was reduced that might be due to the lowering of enzymatic activities at respective conditions.

**Table 4:** ANOVA for response surface quadratic model for fipronil degradation

Source	Sum of squares	df	Mean square	*F* value	*P* value Prob. >*F*	Remarks
Model	8013.8800	9	890.4311	121.4625	<0.0001	Significant
A-X1	335.9232	1	335.9232	45.8228	0.0011	
B-X2	494.5513	1	494.5513	67.4610	0.0004	
C-X3	556.7785	1	556.7785	75.9494	0.0003	
AB	344.1025	1	344.1025	46.9386	0.0010	
AC	3.3489	1	3.3489	0.45682	0.5291	
BC	81.0000	1	81.0000	11.0491	0.0209	
A^2^	3331.5750	1	3331.5750	454.4556	<0.0001	
B^2^	3095.2450	1	3095.2450	422.2182	<0.0001	
C^2^	48.8320	1	48.8320	6.6611	0.0494	
Residual	36.6546	5	7.3309			
Lack of fit	32.6079	3	10.8693	5.3720	0.1609	Not significant
Pure error	4.0467	2	2.0233			
Cor total	8050.5350	14				

*df* degrees of freedom

Previous studies have shown that the application of statistical experimental design techniques in complex processes can result in enhanced yields and allow a rapid and economical determination of the optimum conditions with reduced experiments and minimal resources (Ghevariya et al. 2011; Chen et al. 2013). In the present study, a quadratic polynomial model was effectively used for the optimization of fipronil degradation by strain S1. The optimum values of temperature, pH and inocula biomass for maximum fipronil degradation (85.30 %) were predicted to be 35 °C, 7.5 and 0.175 g L^−1^ from the model. The optimum combinations of variables were validated by conducting experiments and the fipronil degradation of 86.14 % was recorded, which was close to the predicted maximum value.

**Fig. 4:**
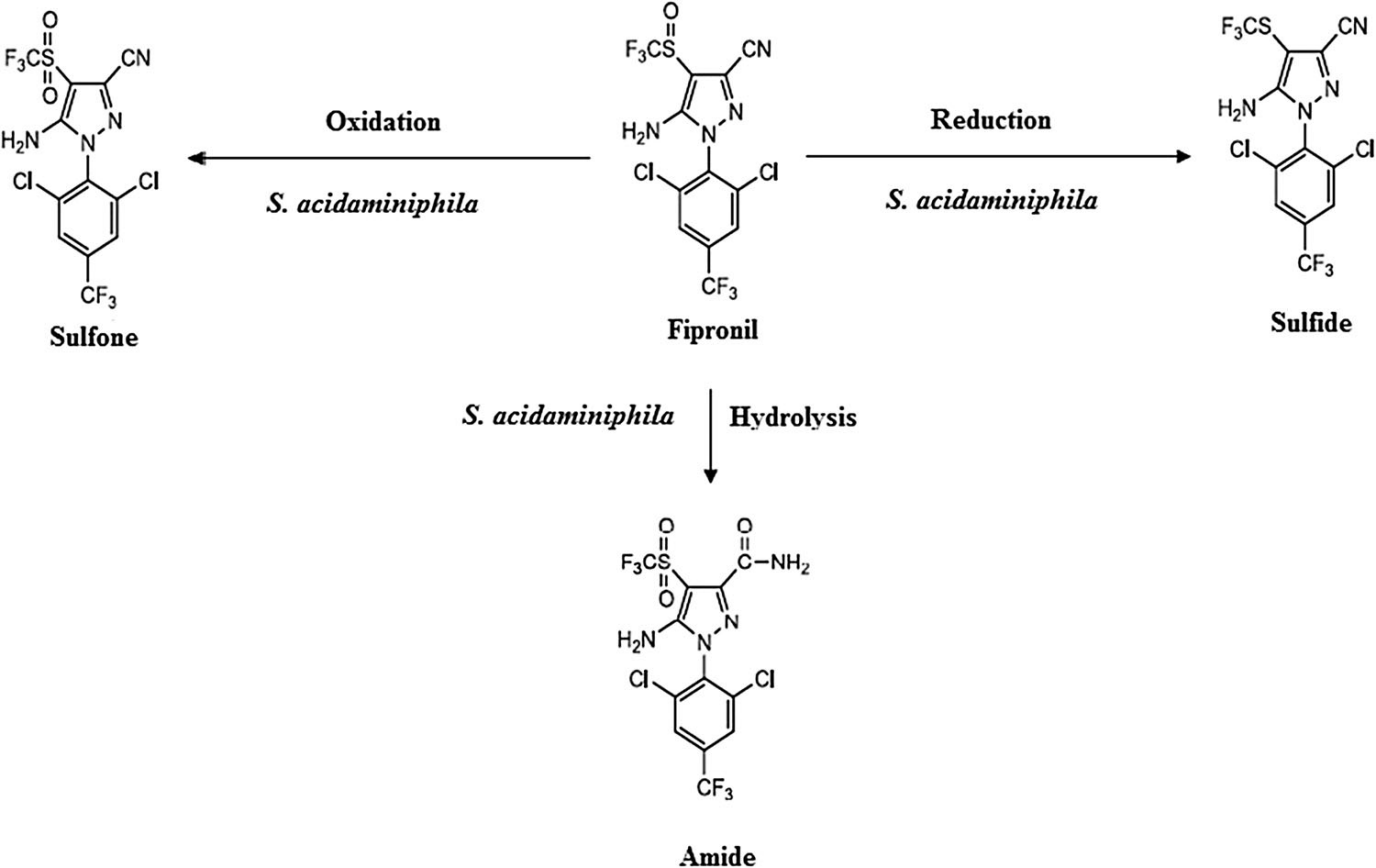
Metabolic pathway for fipronil degradation by *S. acidaminiphila*

The metabolites formed as a result of fipronil degradation were analyzed by GLC. Metabolites formed were identified as fipronil sulfide, sulfone and amide. The retention time of fipronil, fipronil sulfide, fipronil sulfone, fipronil amide was found to be 6.36, 6.14, 8.53 and 11.3 min, respectively. Analyzing the new metabolic products, a novel fipronil degradation pathway for *S. acidaminiphila* was proposed (Fig. 4). The result showed that fipronil was degraded to form sulfone, sulfide and amide by oxidation, reduction and hydrolysis reaction. Although biodegradation of fipronil has been studied (Mandal et al. 2014; Mandal et al. 2013), fipronil degradation pathway by any bacteria is still not reported.

### Persistence of fipronil in sandy loam soil amended with *S. acidaminiphila*

**Fig. 5:**
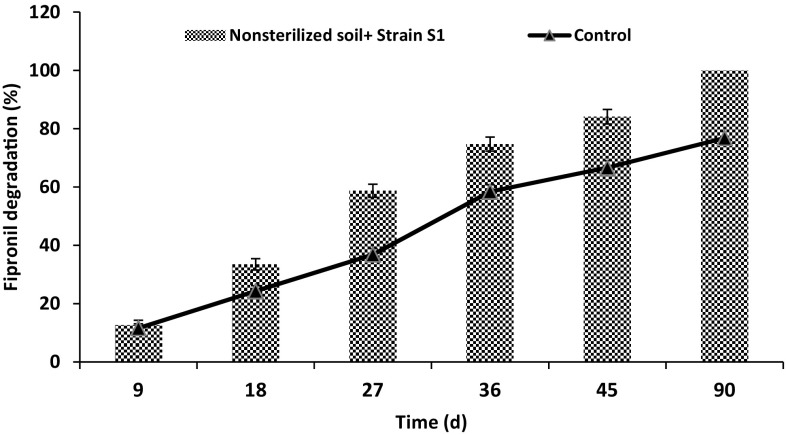
Degradation dynamics of fipronil in non-sterilized soils. Error bars represent the standard deviation within 5 % of the mean

Sandy loam soil fortified with 50 mg kg^−1^ of fipronil was analyzed at 9, 18, 27, 36, 45 and 90 days of time period. Degradation study of fipronil showed significantly greater fipronil degradation (84.12 %) after 45 days of time interval in non-sterilized treatment inoculated with *S.* *acidaminiphila*, however, only 66.64 % fipronil degradation was observed in non-sterilized control condition within the same time interval (Fig. 5). No fipronil residues were detected in non-sterilized treatment after 90 days of time period. Masutti and Mermut (2007) observed significant decline in fipronil levels from 0.689 to 0.399 μg g^−1^ under non-sterile condition within 120 days of incubation period. Similarly, the residues of fipronil were found to persist only up to 10 days in soils fortified with fipronil at the rate of 20 µg kg^−1^ and amended with *Paracoccus* sp. while in the soils fortified at the rate of 80 µg kg^−1^ fipronil, residues persisted up to 20, 30 and 30 days in loamy sand, sandy loam and clay loam, respectively (Kumar et al. 2012).

**Fig. 6:**
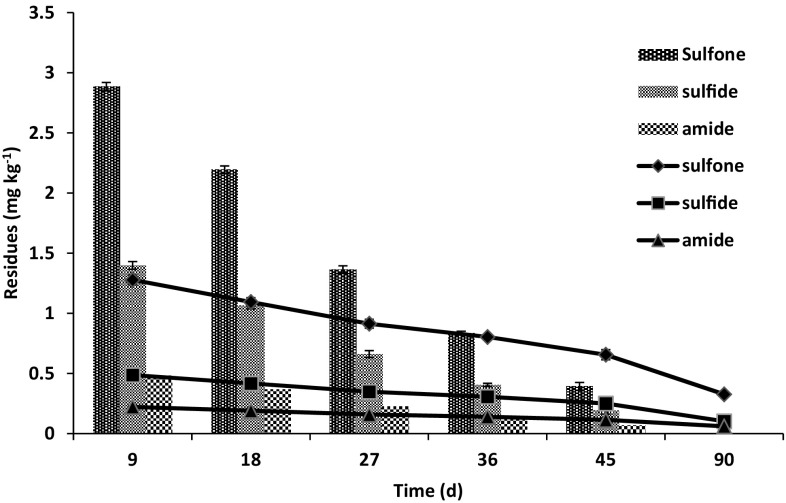
Residues of metabolites (mg kg^−1^) of fipronil degradation in non-sterilized soil inoculated with strain S1 (*bar*) and non-sterilized control (*line*) fortified with 50 mg kg^−1^ fipronil. Error bars represent the standard deviation within 5 % of the mean

The maximum residues of metabolites were found to be 4.76 and 1.98 mg kg^−1^ in soil collected at 7 days, which were further degraded to 0.65 and 1.02 mg kg^−1^ after 45 days in non-sterilized control and non-sterilized treatment, respectively (Fig. 6). Fipronil residues were degraded to below the quantifiable limit of 0.01 mg kg^−1^, in non-sterilized treatment after 90 days of time period. In non-sterilized control, fipronil residues were still present after 90 days of time period with value of 0.66, 0.25 and 0.11 mg kg^−1^ for fipronil sulfone, sulfide and amide, respectively. Mandal et al. (2013) reported quicker degradation of fipronil in *B.* *thuringiensis* inoculated soil. The residues of fipronil and its metabolites were degraded to 0.40 mg kg^−1^ in 21 days after the application of fipronil at the rate of 1.50 mg kg^−1^. Mandal et al. (2014) observed 0.48 mg kg^−1^ residues of fipronil and its metabolites at 28 days after application of fipronil at the rate of 1.50 mg kg^−1^, in soil inoculated with *B. firmus*.

Fipronil sulfone was formed as a major metabolite followed by sulfide and amide (Fig. 5). Ying and Kookana (2002) reported that soil microorganisms accelerated the degradation of fipronil to sulfide and sulfone metabolites while studying fipronil degradation in laboratory and field soil. Masutti and Mermut (2007) found that sulfone derivative (an oxidation product) was the predominant metabolite, whereas the sulfide (a reduction product) derivative was the second most common metabolite found under non-sterile conditions. Sulfone and sulfide concentration increased up to 5 days of incubation, then decreased significantly after 7.5 days. Sulfone values were 34 % lower, while sulfide levels were 31 % higher than those found for the initial 5 days period after 30 days of time period.

### Degradation kinetics of fipronil residues

Fipronil degradation rate in two different conditions was found to follow a pseudo first-order kinetic reaction.

3 $${ \ln }{\raise0.7ex\hbox{${{\text{C}}_{0} }$} \!\mathord{\left/ {\vphantom {{{\text{C}}_{0} } {\text{C}}}}\right.\kern-0pt} \!\lower0.7ex\hbox{${\text{C}}$}} = - K \left( {t - t_{0} } \right)$$

where C_0_ is the maximum concentration of fipronil (mg kg^−1^ in soil); C is the concentration of fipronil (mg kg^−1^) in soil at the time of *t*; *t* is the treatment times in days; *t*_0_ is the treatment time of maximum concentration in days; *k* is fipronil degradation rate constants (days^−1^). The degradation rate constant (*k*) values were calculated from the linear equation obtained from regression plots of ln (C_0_/C) versus time (*t*). Result suggested that the fipronil in non-sterilized treatment inoculated with *S.* *acidaminiphila* degraded faster (equation *C* = 0.77 e^−0.046t^; *R* ^2^ = 0.98; *k* = 0.046; t_1/2_ = 15.09 days) than non-sterilized control (equation *C* = 0.42 e^−0.027^; *R* ^2^ = 0.94; *k* = 0.027; t_1/2_ = 25.67 days). The difference in *k* values and half-life observed in both conditions may be attributed to the fipronil degradation potential of isolated *S.* *acidaminiphila*, which resulted in enhanced degradation. The microbial degradation of fipronil in soils was studied by Zhu et al. (2004) who found that the *t*_1/2_ of fipronil in non-sterile clay loam was 9.72 and 8.78 days at 25 and 35 °C, respectively. Lin et al. (2008) observed that the degradation of fipronil in sediments generally followed exponential decay kinetics and the first-order half-lives of fipronil were only 4.6–18.5 days in anaerobic sediments. Chopra et al. (2010) reported that the dissipation of fipronil residues in soil followed first order kinetics with half life values of 23.35 and 24.31 days at 56 and 112 g a.i. ha^−1^ doses.

## Conclusion

The present study clearly demonstrated the ability of *S.* *acidaminiphila* to degrade and utilize fipronil as a sole source of carbon and energy. To authors’ knowledge, this is the first report of fipronil degradation by *S.* *acidaminiphila*. Under optimum conditions, 86.14 % of fipronil could be degraded after 14 days of cultivation in Dorn’s broth media amended with 25 mg L^−1^ fipronil. Kinetics and fipronil degradation study in non-sterilized soils indicated potential use of *S. acidaminiphila* for bioremediation of fipronil contaminated soil. Inoculation of *S. acidaminiphila* also resulted in complete degradation of toxic metabolites. These findings will certainly provide new insights in the indication of useful ways for bioremediation of fipronil-contaminated environment.
